# Prevalence and Risk Factors of Metabolic Dysfunction–Associated Steatotic Liver Disease in Patients With Type 2 Diabetes Mellitus at a Tertiary Center in Saudi Arabia: Cross-Sectional Questionnaire Study

**DOI:** 10.2196/77772

**Published:** 2025-11-03

**Authors:** Abdulrahman M Elnasieh, Mohammed Almesned, Akram N Al Hazmi, Atheer Alturki, Faisal I Alhawaidi, Razan K Alhadlq, Maryam Alramadhan, Nasser Alobilan, Yasser Sheikh Qroosh

**Affiliations:** 1 Family Medicine, King Saud Medical City, P.O Box 2897, Riyadh, 11196, Saudi Arabia, 966 50 371 6325; 2 King Saud Medical City, Riyadh, Saudi Arabia; 3 Radiology, King Saud Medical City, Riyadh, Saudi Arabia

**Keywords:** non-alcoholic fatty liver, type 2 diabetes mellitus, biochemical markers, MASLD, insulin resistance, metabolic dysfunction–associated steatotic liver disease

## Abstract

**Background:**

The escalating rates of obesity and type 2 diabetes mellitus (T2DM) in Saudi Arabia highlight the impending burden of metabolic dysfunction–associated steatotic liver disease (MASLD) and nonalcoholic steatohepatitis.

**Objective:**

This study aimed to identify MASLD among patients with T2DM at King Saud Medical City family medicine clinics, Riyadh, and explore associated factors to facilitate early intervention and prevention strategies.

**Methods:**

This cross-sectional study identified patients with T2DM who attended King Saud Medical City, Riyadh, underwent an abdominal ultrasound, and were diagnosed with MASLD. The study data were collected by a peer-reviewed validated data extraction sheet and analyzed by SPSS (version 26.0; IBM Corp).

**Results:**

Our study included 292 participants, with 47.3% (n=138) males and 52.7% (n=154) females. Notably, the prevalence of MASLD was 54.5% (n=159). Prevalent comorbidities included dyslipidemia (218/292, 74.7%) and hypertension (209/292, 71.6%). Most participants were nonsmokers (218/292, 74.7%). Higher waist circumference was significantly associated with MASLD (*P*=.02), with >80 cm among females (85/141, 60.3%) and >94 cm among males (60/141, 54.5%) affected across different stages of MASLD. Obesity (BMI>30 kg/m^2^) also significantly correlated with MASLD (*P*<.001). Individuals taking aspirin had half the odds of MASLD development (odds ratio [OR] 0.523, 95% CI 0.331-0.844; *P*=.007). Biochemical analysis revealed significant associations between MASLD and elevated alanine aminotransferase (*P*=.009), aspartate aminotransferase (*P*=.01), and homeostatic model assessment of insulin resistance (*P*=.001). Total cholesterol (*P*=.01), triglycerides (*P*=.03), and low-density lipoprotein (*P*=.04) were significantly elevated in patients with MASLD. Insulin exhibited a significant positive correlation with MASLD (*r*=0.24; *P*=.001). Glucose levels showed no significant association (*r*=0.03; *P*=.63).

**Conclusions:**

Our study highlights significant associations between MASLD and various factors, including waist circumference, obesity, and certain biochemical markers. Furthermore, the protective effect of aspirin against MASLD warrants further investigation. These findings underscore the importance of early intervention and targeted preventive strategies.

## Introduction

In the last quarter of 2023, the American Association for the Study of Liver Diseases, the European Association for the Study of the Liver, and the Sur Medical Clinic Foundation jointly released a Delphi consensus statement [1]. These organizations recognized the significant limitations of the current terms, nonalcoholic fatty liver disease (NAFLD) and nonalcoholic steatohepatitis (NASH). As a result, a new nomenclature for fatty liver disease was introduced and published in the *Journal of Hepatology* [2]. The term chosen to replace NAFLD is metabolic dysfunction–associated steatotic liver disease (MASLD) [2]. The changes to the previous terminology will be integrated into this research compilation, while maintaining the original terms as referenced. In addition, the new nomenclature, acronym, and diagnostic criteria received broad support and are expected to enhance awareness and patient identification [2].

Even though NASH was first used in 1989, it required another 10 years for its inclusion in the clinicopathologic spectrum of MASLD, which includes subtypes with varying likelihood for progression [3]. The term MASLD refers to the buildup of fat in the liver, specifically when the amount of fat in the liver exceeds 5% of the hepatocytes [4]. In addition, MASLD is characterized by progressive steatosis and related pathology, such as cirrhosis, hepatitis, or hepatocellular carcinoma. Patients with MASLD can display a diverse range of histological features, including simple steatosis, NALFD, or NASH [5]. Over the years, the prevalence of chronic liver diseases has risen, leading to concerning increases in liver-related morbidity and mortality rates globally [6]. MASLD is one of the primary contributors to chronic liver diseases and has escalated to a worldwide epidemic, impacting approximately 1 in 4 adults, with an estimated prevalence of 25% to 30% [6,7]. This trend seems to correlate with the rising incidence of metabolic syndrome and its associated factors, such as obesity, type 2 diabetes mellitus (T2DM), and dyslipidemia [6].

The diagnostic criteria of MASLD include invasive and noninvasive tests. Noninvasive fibrosis tests should be used to rule out advanced fibrosis in low-prevalence populations and patients at risk of advanced liver fibrosis, such as those with metabolic risk factors or harmful alcohol use. These tests are part of routine investigations in primary care for suspected liver disease. Automatic calculation and systematic reporting of simple noninvasive tests, such as Fibrosis-4, is recommended to improve risk stratification and care linkage [8]. However, liver biopsy continues to be the most reliable method for diagnosing this condition. However, there are a variety of noninvasive methods, such as imaging tests and biomarkers, that are becoming more widely used for the assessment of fibrosis [9].

In Saudi Arabia, the prevalence of MASLD is expected to increase through 2030, parallel to projected increases in the prevalence of obesity and DM. By 2030, there would be a pre-estimated 12,534,000 MASLD cases in Saudi Arabia [10].

It is assumed that 30% of the people aged ≥15 years in 2015 had MASLD. The prevalence of MASLD among older adults in Saudi Arabia is estimated at 24.8% [10]. The prevalence of MASLD continues to rise and is now becoming one of the most frequent causes of cirrhosis (advanced liver disease) and liver transplantation in Europe [11].

Unless patients with MASLD are identified and diagnosed, they are denied the knowledge and opportunity to make the necessary changes. It is important to identify and risk-stratify patients with MASLD in order to implement therapeutic interventions [11]. The latter statements are the major incentives to initiate this research to identify patients with MASLD among T2DM attending King Saud Medical City (KSMC), Riyadh. Patients with MASLD have an increased risk of dying from liver disease, cardiovascular disease, and most causes of cancer, with modeling suggesting that the annual predicted economic burden of MASLD in Europe would be more than €35 billion (approximately US $40) of direct cost and further €200 billion of societal cost [12]. Primary care practitioners are central to the management of patients with NAFLD, but data on the knowledge and attitude of primary care practitioners toward MASLD are lacking. Future guidelines should emphasize strategies for screening vulnerable populations (obese and diabetics) [13].

It has been emphasized in recent relevant literature that MASLD is strongly linked with an unhealthy lifestyle. This is an area where improvement strategies could be designed and implemented in communities, including schools. Another important drive to conduct this research is the close relationship between MASLD and T2DM, and the management of the latter will go far in arresting the progression of MASLD. Family medicine and primary care practitioners are in a central position to provide timely screening and early diagnosis of MASLD and possible reduction in the future prevalence rates, as these practitioners serve as gatekeepers and are the primary health care professionals providing most of the care for these patients. The main aim of this study is to identify patients with T2DM who have MASLD attending KSMC, Riyadh, under the care of Family Medicine Department health care professionals.

## Methods

### Study Design, Setting, and Time Frame

This is an analytical, descriptive, observational, cross-sectional situational analysis survey that identified patients with T2DM, reviewed whether they had a recent ultrasound liver imaging, and ordered this imaging modality for those who are included in the study. The diagnostic criteria of MASLD in this study were based on ultrasound live imaging [4]. The study was conducted at KSMC Riyadh during the study period, as per the research work plan.

### Sample Size Calculation

The sample size was calculated using the formula *z*∝/1(*P*)(1−*P*) 2 *d*2, where *z*=1.96 (corresponding to a 95% CI value) and the *P* (the reported prevalence of MASLD among patients with type 2 diabetes in a study conducted in Abha, Saudi Arabia, in 2018) is 72.7% [14]. While the margin of error was indicated as 5%, the final sample size was 309.72. To control for the low response rate, the sample size was increased by 10%. Using Epi-Info with the sample data, the initial sample size was calculated as 310, resulting in a final sample size of 341.

### Sampling Method and Participants

The sampling method was a convenience sampling technique, and the study identified patients with T2DM from the pool of KSMC Riyadh software data. The sample included all the patients who satisfied the criteria for inclusion in the study. The source of the data was the Employee Health Clinic, Chronic Disease Services, and Chronic Disease Refill Clinic. Those who had MASLD reported from a recent radiological order were included in the study, ideally within the last 12 months. The included participants were contacted to obtain their consent to be included in the study sample. This process entailed accessing their electronic medical records and reviewing the data available.

### Eligibility Criteria

The inclusion and exclusion criteria are listed in Textbox 1.

Textbox 1. **Inclusion and exclusion criteria.**

**Inclusion criteria**

- All patients with type 2 diabetes mellitus who were more than 30 years of age, regardless of both genders.
- All the patients included must have had a liver ultrasound image within 1 year.
- Patients attending the above-named Family Medicine Clinics.

**Exclusion criteria**

- All pregnant ladies are under obstetrics and gynecology care.
- All patients with type 1 diabetes mellitus.
- All patients with type 2 diabetes mellitus undergoing dialysis.
- All patients with other liver diseases (eg, viral hepatitis and alcohol use).

#### Data Collection Tool

The study data were extracted from the KSMC database by investigators. The data extraction tool or questionnaire was prepared by collating tools from previous studies, which were adapted by the investigators. This questionnaire (Multimedia Appendix 1) had the following sections:

1. Characteristics of the study subjects (age, gender, marital status, educational level, nationality, medications, vaccination history, duration of T2DM, and smoking history).
2. Anthropometric, biochemical, and ultrasound characteristics (BMI, waist circumference [WC], and blood pressure), lipid profile, liver enzyme level, and hepatic steatosis, identified on ultrasound image and other laboratory indices as stated in the data collection sheet.
3. Associated comorbidities as mentioned in the questionnaire or data extraction tool.

#### Validity

Face validity of the questionnaire was conducted by expert researchers to ensure the questions were clear and understandable for participants. This process involved reviewing the wording, format, and overall structure of the questionnaire to confirm its appropriateness for the target population. In addition, feedback from pilot testing was used to make any necessary revisions before final administration. The content validity of the questionnaire was assessed through expert review and the use of established measurement techniques. The questionnaire was ultimately deemed appropriate for the target population.

#### Data Variables

The variables to be measured during the study included dependent and independent variables. The former refers to the diseases to be studied, namely MASLD among patients with T2DM, which represents the outcome. The latter refers to the variables encompassing risk factors that influence the outcome, also called predictors, such as associated comorbidities, anthropometric characteristics, and biochemical indicators. Another detailed classification of the variables and their subdivisions included categorical or qualitative variables. The nominal categorical variables included nationality, marital status, and medications used by the patients. The binary qualitative variable will include gender and vaccination history. The ordinal categorical variables included the educational level and smoking status. The quantitative numerical variables included continuous subgroups such as age, weight, BMI, and laboratory parameters.

### Statistical Analysis

SPSS (version 26.0; IBM Corp) was used to analyze the data. For categorical variables, the results were displayed as numbers and percentages; for continuous variables (such as insulin and glucose levels), they were displayed as mean and SD. Pearson chi-square was used to ascertain whether MASLD and other factors have a significant association. The effects of significant variables, such as gender, WC, obesity, alanine aminotransferase (ALT) and aspartate aminotransferase (AST) levels, homeostatic model assessment of insulin resistance (HOMA-IR), cholesterol-lowering medications, and aspirin use, on the risk of MASLD were determined using a logistic regression model. Statistical significance was defined as a *P*<.05.

### Ethical Considerations

The study was approved by the Institutional Review Board of King Saud Medical City (protocol code H1RI-07-Mar23-01 and date of approval was May 14, 2023). After identifying the patients to be enrolled in the study, they were contacted by the investigators through the KSMC Riyadh central telephone system, and verbal informed consent was taken from all participants. The researchers ensured the anonymity of the identities of participants. Confidentiality of the collected data and possible publication of the study were acknowledged by the investigators.

## Results

The overall prevalence of MASLD in our study population was 54.5% (n=159). Table 1 provides an overview of the sociodemographic and clinical characteristics of the participants in the study, which included 292 individuals. The total response rate was 85%. Of 292 participants, there were 138 (47.3%) males and 154 (52.7%) females. Regarding age groups, the majority of participants fell within the 41‐59 years category (192/292, 65.8%), followed by those aged 60 years and older (94/292, 32.2%), with a smaller proportion in the 30‐40 years group (6/292, 2.1%). The vast majority of participants were married (243/292, 83.2%) and had received at least an elementary education (79/292, 27.1%). In terms of physical activity, 30.8% (90/292) reported being physically active. The study population exhibited various comorbidities, with dyslipidemia being the most prevalent (218/292, 74.7%), followed by hypertension (209/292, 71.6%). Other comorbidities included cardiac disease (108/292, 37%), hypothyroidism (32/292, 11%), and bronchial asthma (19/292, 6.5%). The duration of diabetes was relatively evenly distributed, with 28.4% (83/292) having diabetes for less than 5 years and 71.6% (209/292) for 5 years or more. In terms of current treatment for diabetes, the majority were on oral hypoglycemics only (179/292, 61.3%), followed by a combination of oral hypoglycemics and insulin (92/292, 31.5%). In addition, most participants were taking antihypertensive medications (216/292, 74%) and lipid-lowering agents (226/292, 77.4%). However, only 46.9% (137/292) were taking aspirin. The majority of participants were nonsmokers (218/292, 74.7%), with a smaller proportion being current smokers (30/292, 10.3%) or ex-smokers (44/292, 15.1%). Regarding WC, a considerable proportion of males (110/292, 37.7%) and females (141/292, 48.3%) exceeded the recommended thresholds of 94 cm and 80 cm, respectively. Furthermore, the distribution of BMI classifications varied, with the majority falling within the overweight and obese categories (BMI 25‐39.9 kg/m²).

**Table 1.** Sociodemographic and clinical characteristics of participants (N=292).

Characteristics	Values (N=292), n (%)
Sex	
Male	138 (47.3)
Female	154 (52.7)
Age (years)	
30-40	6 (2.1)
41-59	192 (65.8)
≥60	94 (32.2)
Marital status	
Unmarried	11 (3.8)
Married	243 (83.2)
Divorced	20 (6.8)
Widow or widower	18 (6.2)
Duration of diabetes (years)	
<5	83 (28.4)
≥5	209 (71.6)
Current treatment of DM^a^	
Oral hypoglycemics only	179 (61.3)
Oral hypoglycemic+insulin	92 (31.5)
Insulin only	18 (6.2)
Diet and exercise only	1 (0.3)
Others	2 (0.7)
Antihypertensive medication	
Yes	216 (74)
No	76 (26)
Lipid-lowering agents	
Yes	226 (77.4)
No	66 (22.6)
Aspirin	
Yes	137 (46.9)
No	155 (53.1)
Smoking	
Nonsmoker	218 (74.7)
Current smoker	30 (10.3)
Ex-smoker	44 (15.1)
Waist circumference measurement (cm)	
Males	
<94	30 (10.3)
>94	110 (37.7)
Females	
<80	11 (3.8)
>80	141 (48.3)
BMI classification (kg/m^2^)	
<18	1 (0.3)
18.5-24.9	38 (13)
25-29.9	91 (31.2)
30-34.9	99 (33.9)
35-39.9	33 (11.3)
40 and more	30 (10.3)

a DM: diabetes mellitus.

Table 2 presents the laboratory findings of the participants in the study. The majority of participants (251/292, 86%) had normal ALT levels, while a smaller percentage (34/292, 11.6%) had high ALT levels. As for AST, most participants (273/292, 93.5%) had normal levels, with a small proportion (17/292, 5.8%) having high levels. For gamma-glutamyl transferase (GGT), the majority had normal levels (177/292, 60.6%), while some had high levels (22/292, 7.5%). In terms of glycated hemoglobin (HbA_1c_) levels, the largest proportion fell within the range of 6.4‐10.0 (173/292, 59.2%), followed by >10 (55/292, 18.8%), indicating suboptimal glycemic control in a significant portion of the participants. Regarding triglyceride levels, most participants had normal levels (197/292, 67.5%), while a smaller percentage had borderline high (44/292, 15.1%) or high levels (46/292, 15.8%). Similarly, for low-density lipoprotein (LDL), the majority had normal or optimal levels (184/292, 63%), with smaller percentages in the borderline high, high, and very high categories. Vitamin D levels varied, with a considerable portion of participants having either deficiency (104/292, 35.6%) or insufficiency (71/292, 24.3%). Vitamin B_12_ levels and glucose levels were mostly high (201/292, 68.8% both). Insulin levels were also predominantly normal (171/292, 58.6%), with a notable percentage of participants having high levels (28/292, 9.6%). The HOMA-IR indicated that nearly half of the participants had high insulin resistance (143/292, 49%). Ultrasound reports revealed that the majority of participants had either normal or unremarkable findings (133/292, 45.5%), while 54.5% (159/292) were diagnosed with MASLD based on ultrasound reports.

**Table 2.** Biochemical findings of the participants in the study.

Characteristics	Values (N=292)
ALT^a^, n (%)	
Normal	251 (86)
Low	5 (1.7)
High	34 (11.6)
Not available	2 (0.7)
AST^b^, n (%)	
Normal	273 (93.5)
High	17 (5.8)
Not available	2 (0.7)
GGT^c^, n (%)	
Normal	177 (60.6)
High	22 (7.5)
Not available	93 (31.8)
HbA_1c_^d^, n (%)	
<5.9	20 (6.8)
5.9-6.4	42 (14.4)
6.4-10	173 (59.2)
>10	55 (18.8)
Not available	2 (0.7)
Triglycerides, n (%)	
Normal	197 (67.5)
Borderline high	44 (15.1)
High	46 (15.8)
Very high	2 (0.7)
Not available	3 (1)
LDL^e^, n (%)	
Normal or optimal	184 (63)
Near optimal	42 (14.4)
Borderline high	30 (10.3)
High	25 (9.6)
Very high	8 (2.7)
Not available	3 (1)
Vitamin D, n (%)	
Deficiency	104 (35.6)
Insufficiency	71 (24.3)
Normal	61 (20.9)
High	29 (9.9)
Not available	27 (9.2)
Vitamin B_12_, n (%)	
Low	6 (2.1)
Normal	71 (24.3)
High	201 (68.8)
Not available	14 (4.8)
Glucose, mean (SD)	8.53 (3.7)
Low, n (%)	6 (2.1)
Normal, n (%)	71 (24.3)
High, n (%)	201 (68.8)
Not available, n (%)	14 (4.8)
Insulin, mean (SD)	95.59 (79.3)
Low, n (%)	6 (2.1)
Normal, n (%)	171 (58.6)
High, n (%)	28 (9.6)
Not available, n (%)	87 (29.8)
HOMA-IR^f^, n (%)	
Low	5 (1.7)
Normal	47 (16.1)
High	143 (49)
Not available	97 (33.2)
Ultrasound report, n (%)	
Normal or unremarkable	133 (45.5)
Fatty liver grade I	82 (28.1)
Fatty liver grade II	66 (22.6)
Fatty liver grade III	8 (2.7)
Cirrhosis	3 (1)
MASLD^g^ prevalence based on ultrasound reports, n (%)	
Yes	159 (54.5)
No	133 (45.5)

a ALT: alanine aminotransferase.

b AST: aspartate aminotransferase.

c GGT: gamma-glutamyl transferase.

d HbA_1c_: glycated hemoglobin.

e LDL: low-density lipoprotein.

f HOMA-IR: homeostatic model assessment of insulin resistance.

g MASLD: metabolic dysfunction–associated steatotic liver disease.

Table 3 presents the association using the chi-square test between sociodemographic and clinical characteristics and MASLD stages among the study participants. There were no significant associations observed between MASLD stages and age groups, gender, physical activity, hypertension, dyslipidemia, hypothyroidism, bronchial asthma, cardiac disease, stroke, epilepsy, depression, kidney disease, treatment of diabetes mellitus, or antihypertensive medications. However, a significant association was found between high WC and MASLD, with 60.3% (85/141) of females and 54.5% (60/110) of males with WC greater than the normal range having MASLD (*P*=.02), as well as a significant association regardless of gender (*P*=.003). In addition, obesity (BMI >30 kg/m²) was significantly associated with MASLD (*P*<.001), as well as increased BMI, where higher BMI categories showed higher prevalence of MASLD (*P*<.001). These findings suggest that factors such as WC, obesity, and BMI may play crucial roles in the development of MASLD among patients with T2DM.

**Table 3.** Association of sociodemographic and clinical characteristics and stages of metabolic dysfunction–associated steatotic liver disease.

Variables		Stages, n (%)					*P* value
		No MASLD^a^	1	2	3	4	
Age (years)							.23
30-40		4 (66.7)	0 (0)	2 (33.3)	0 (0)	0 (0)	
41-59		78 (40.6)	60 (31.3)	47 (24.5)	6 (3.1)	1 (0.5)	
>60		50 (53.2)	23 (24.5)	17 (18.1)	2 (2.1)	2 (2.1)	
Sex							.01
Female		59 (38.3)	52 (33.8)	36 (23.4)	5 (3.2)	2 (1.3)	
Male		73 (52.9)	31 (22.5)	30 (21.7)	3 (2.2)	1 (0.7)	
Physically active							.80
No		91 (45)	54 (26.7)	49 (24.3)	6 (3)	2 (1)	
Yes		41 (45.6)	29 (32.2)	17 (18.9)	2 (2.2)	1 (1.1)	
Waist circumference (cm)							
Female							.022^b^^,^^c^
<80		4 (36.4)	3 (27.3)	3 (27.3)	0 (0)	1 (9.1)	
>80		56 (39.7)	47 (33.3)	33 (23.4)	4 (2.8)	1 (0.7)	
Male							.022^b^^,^^c^
<94		22 (73.3)	4 (13.3)	3 (10)	0 (0)	1 (3.3)	
>94		50 (45.5)	29 (26.4)	27 (24.5)	4 (3.6)	0 (0)	
BMI (kg/m^2^)							<.001^b^^,^^c^
<18		1 (100)	0 (0)	0 (0)	0 (0)	0 (0)	
18.5‐24.9		24 (63.2)	7 (18.4)	5 (13.2)	1 (2.6)	1 (2.6)	
25‐29.9		59 64.8	20 (22)	11 (12.1)	0 (0)	1 (1.1)	
30‐34.9		34 (34.3)	34 (34.3)	28 (28.3)	2 (2)	1 (1)	
35‐39.9		8 (24.2)	11 (33.3)	12 (36.4)	2 (6.1)	0 (0)	
>40		6 (20)	11 (36.7)	10 (33.3)	3 (10)	0 (0)	

a MASLD: metabolic dysfunction–associated steatotic liver disease.

b *P *values were calculated by chi-square test.

c *P*<.05 denotes statistical significance.

Table 4 provides insights into the association using the chi-square test between biochemical findings and MASLD stages among the study participants. High levels of ALT were significantly associated with MASLD, with 41.2% (14/34) of participants in stage 2 having high ALT levels. Similarly, high AST levels were significantly associated with MASLD, with 29.4% (5/17) of participants in stage 2, and 11.8% (2/17) in stage 4 having high AST levels. However, there was no significant association between GGT levels and MASLD. Regarding insulin resistance markers, high HOMA-IR levels were significantly associated with MASLD, with 31.5% (45/143) of participants in stage 2 having high HOMA-IR levels. However, no significant associations were found between MASLD and HbA_1c_ levels, total cholesterol, triglycerides, albumin, LDL levels, vitamin D levels, and vitamin B_12_ levels. When comparing the mean (SD) values of glucose and insulin according to stages, no significant differences were observed with glucose levels according to MASLD stages (*P*=.69), whereas significantly higher insulin levels were observed among patients with stage 2 and 3 compared with patients without MASLD (*P*=.008).

**Table 4.** Association of biochemical findings and MASLD stages. “Not available” values were excluded from the analysis.

Biomarker^a^		Stages					*P* value
		No MASLD^b^	1	2	3	4	
ALT^c^							.009^d^
High		9 (26.5)	8 (23.5)	14 (41.2)	1 (2.9)	2 (5.9)	
Low		2 (40)	3 (60)	0 (0)	0 (0)	0 (0)	
Normal		121 (48.2)	70 (27.9)	52 (20.7)	7 (2.8)	1 (0.4)	
AST^e^							.01^d^^,^^f^
High		5 (29.4)	4 (23.5)	5 (29.4)	1 (5.9)	2 (11.8)	
Normal		127 (46.5)	77 (28.2)	61 (22.3)	7 (2.6)	1 (0.4)	
GGT^g^							.38
High		7 (31.8)	5 (22.7)	9 (40.9)	1 (4.5)	0 (0)	
Normal		82 (46.3)	47 (26.6)	40 (22.6)	6 (3.4)	2 (1.1)	
HbA_1c_^h^							.78
<5.9		13 (65)	3 (15)	4 (20)	0 (0)	0 (0)	
5.9‐6.4		22 (52.4)	8 (19)	10 (23.8)	1 (2.4)	1 (2.4)	
6.4‐10		71 (41)	54 (31.2)	41 (23.7)	6 (3.5)	1 (0.6)	
>10		26 (47.3)	17 (30.9)	10 (18.2)	1 (1.8)	1 (1.8)	
Total cholesterol							.06
Borderline		10 (24.4)	11 (26.8)	19 (46.3)	1 (2.4)	0 (0)	
Desirable		106 (50.2)	56 (26.5)	40 (19)	6 (2.8)	3 (1.4)	
High		11 (36.7)	14 (46.7)	4 (13.3)	1 (3.3)	0 (0)	
Low		3 (60)	0 (0)	2 (40)	0 (0)	0 (0)	
Normal		0 (0)	0 (0)	1 (100)	0 (0)	0 (0)	
Triglyceride							.45
Borderline high		16 (36.4)	13 (29.5)	12 (27.3)	3 (6.8)	0 (0)	
High		15 (32.6)	17 (37)	13 (28.3)	1 (2.2)	0 (0)	
Normal		98 (49.7)	51 (25.9)	41 (20.8)	4 (2)	3 (1.5)	
Very high		1 (50)	1 (50)	0 (0)	0 (0)	0 (0)	
LDL^i^							.88
Borderline high		8 (26.7)	12 (40)	8 (26.7)	2 (6.7)	0 (0)	
High		7 (28)	8 (32)	9 (36)	1 (4)	0 (0)	
Near optimal		22 (52.4)	12 (28.6)	7 (16.7)	0 (0)	1 (2.4)	
Normal		2 (50)	1 (25)	1 (25)	0 (0)	0 (0)	
Not available		2 (66.7)	1 (33.3)	0 (0)	0 (0)	0 (0)	
Optimal		88 (48.9)	46 (25.6)	39 (21.7)	5 (2.8)	2 (1.1)	
Very high		3 (37.5)	3 (37.5)	2 (25)	0 (0)	0 (0)	
Vitamin D							.93
Deficiency		49 (47.1)	30 (28.8)	21 (20.2)	3 (2.9)	1 (1)	
High>100		11 (37.9)	7 (24.1)	10 (34.5)	1 (3.4)	0 (0)	
Insufficiency		32 (45.1)	18 (25.4)	18 (25.4)	2 (2.8)	1 (1.4)	
Normal level		26 (42.6)	21 (34.4)	12 (19.7)	2 (3.3)	0 (0)	
Insulin							.27
High		8 (28.6)	8 (28.6)	11 (39.3)	1 (3.6)	0 (0)	
Low		5 (83.3)	1 (16.7)	0 (0)	0 (0)	0 (0)	
Normal		73 (42.7)	49 (28.7)	43 (25.1)	4 (2.3)	2 (1.2)	
Not available		41 (56.9)	18 (25)	10 (13.9)	2 (2.8)	1 (1.4)	
HOMA-IR^j^							<.001^d^^,^^f^
High		43 (30.1)	48 (33.6)	45 (31.5)	5 (3.5)	2 (1.4)	
Low		3 (60)	2 (40)	0 (0)	0 (0)	0 (0)	
Normal		36 (76.6)	5 (10.6)	6 (12.8)	0 (0)	0 (0)	

a Total may vary from 292, since analysis was done on patients with results only.

b MASLD: metabolic dysfunction–associated steatotic liver disease.

c ALT: alanine aminotransferase.

d *P *values calculated by chi-square test.

e AST: aspartate aminotransferase.

f *P*<.05 denotes statistical significance.

g GGT: gamma-glutamyl transferase.

h HbA_1c_: glycated hemoglobin.

i LDL: low-density lipoprotein.

j HOMA-IR: homeostatic model assessment of insulin resistance.

Table 5 provides insights into the association between sociodemographic characteristics and MASLD regardless of severity among the study participants. Variables including age groups, marital status, and educational level did not show significant associations with MASLD. However, when grouped by sex, females showed a significantly higher prevalence of MASLD compared with males (94/154, 61% vs n=65/138, 47.1%). Similarly, there were no significant associations between MASLD and physical activity. However, a significantly higher proportion of patients with high WC have MASLD (146/253, 57.7%) compared with those with normal WC (13/39, 33.3%). In addition, BMI showed a significant association with MASLD, with higher proportions of MASLD observed among patients with BMI 30-34.9 kg/m² (64/99, 64.6%), 35‐39.9 kg/m² (25/33, 75.8%), and >40 kg/m² (24/30, 80%).

**Table 5.** Association of sociodemographic characteristics and metabolic dysfunction–associated steatotic liver disease. “Not available” values were excluded from the analysis.

Characteristics^a^		MASLD^b^		*P* value^c^
		No MASLD	With MASLD	
Age (years)				.05
30‐40		4 (66.7)	2 (33.3)	
41‐59		78 (40.6)	114 (59.4)	
>60		51 (54.3)	43 (45.7)	
Sex				.017^d^
Female		60 (39)	94 (61)	
Male		73 (52.9)	65 (47.1)	
Physically active				>.99
No		92 (45.5)	110 (54.5)	
Yes		41 (45.6)	49 (54.4)	
Waist circumference (cm)				.004^d^
Normal		26 (66.7)	13 (33.3)	
High		107 (42.3)	146 (57.7)	
BMI (kg/m^2^)				<.001^d^
<18		1 (100)	0 (0)	
18.5-24.9		24 (63.2)	14 (36.8)	
25-29.9		59 (64.8)	32 (35.2)	
30-34.9		35 (35.4)	64 (64.6)	
35-39.9		8 (24.2)	25 (75.8)	
>40		6 (20)	24 (80)	

a Total may vary from 292, since analysis was done on patients with results only.

b MASLD: metabolic dysfunction–associated steatotic liver disease.

c *P* values calculated by chi-square test.

d *P*<.05 denotes statistical significance.

**Figure 1.**
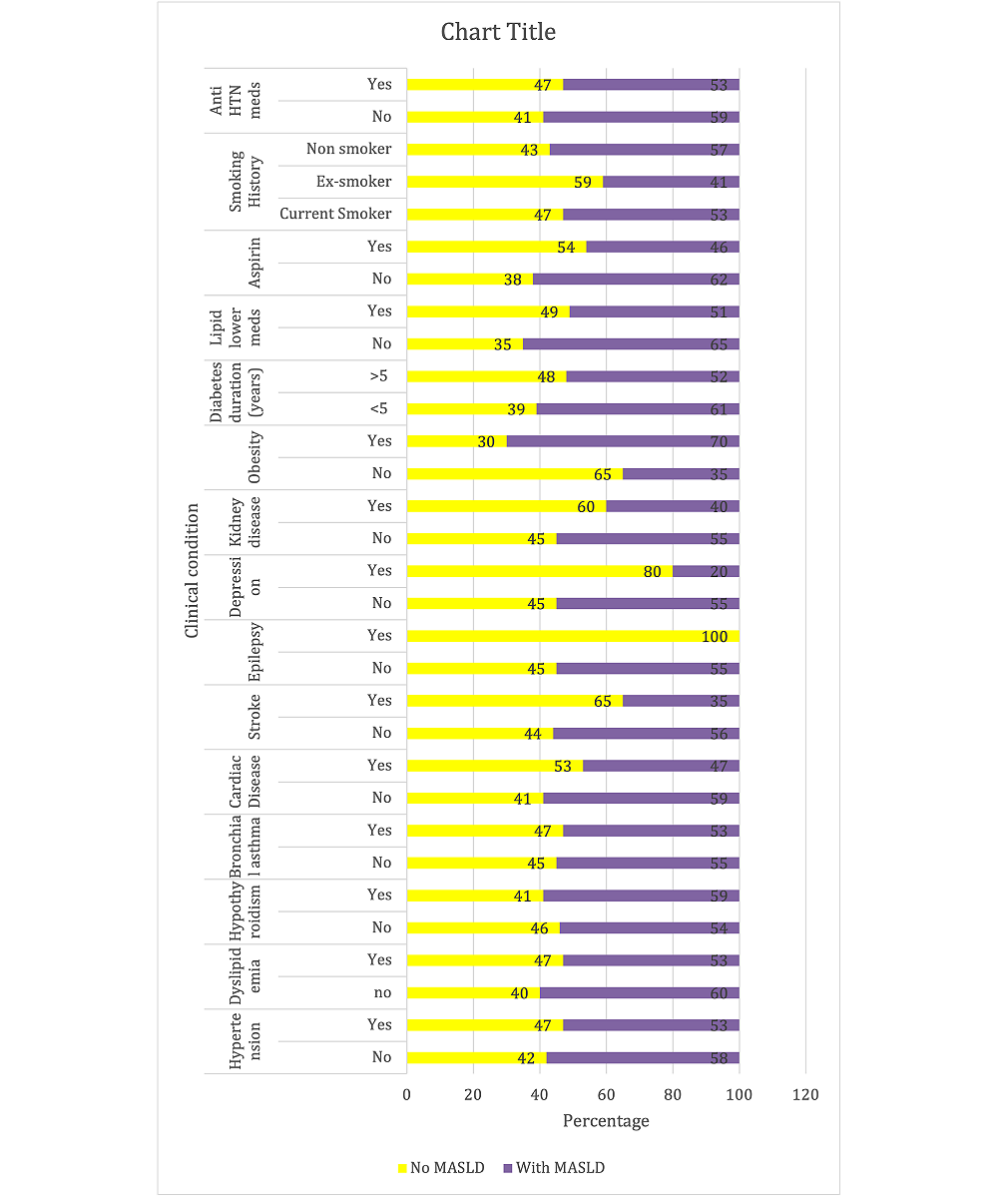
Association of clinical characteristics and metabolic dysfunction-associated steatotic liver disease.

Figure 1 shows the relationship between various clinical characteristics and MASLD among the study participants. Results indicate no significant association between MASLD and hypertension, dyslipidemia, hypothyroidism, bronchial asthma, cardiac disease, stroke, epilepsy, depression, kidney disease, and smoking history. However, there was a significant association between MASLD and obesity (*P*<.001), with a higher proportion of patients with obesity (113/162, 69.8%) exhibiting MASLD compared with those without obesity (46/130, 35.4%). In addition, patients on lipid-lowering medications (*P*=.047) and those taking aspirin (*P*=.006) showed significant associations with MASLD, with higher proportions of MASLD among those not taking these medications. Furthermore, no significant association was found between MASLD and the duration of diabetes or the use of antihypertensive medications.

Categorical variables were represented as numerical variables, and collinearity diagnostics were performed by calculating the variance inflation factor and tolerance. Variance inflation factor for ALT was 1.36, AST was 1.36, HOMA-IR was 1.04, lipid-lowering drugs was 1.14, aspirin was 1.16, gender was 1.11, and WC was 1.08. This signifies that there was a moderate correlation, but it was not severe enough to warrant corrective measures.

Table 6 shows the series of sequential logistic regression models. Unadjusted logistic regression showed a significantly increased risk associated with male sex, high WC, presence of obesity, high ALT, and high HOMA-IR on MASLD, and with a significantly decreased risk associated with MASLD for patients who used insulin (refer to OR in Table 6). When adjusted for age, gender, and obesity, high ALT and high HOMA-IR remained to significantly increase the risk for MASLD (model 2). When adjusted for age, gender, obesity, and the use of lipid-lowering drugs and aspirin, high ALT and high HOMA-IR remained significant risk factors for MASLD (model 3).

**Table 6.** Series of sequential logistic regressions of risk factors for metabolic dysfunction–associated steatotic liver disease.

Factors	UOR^a^ (95% CI) Model 1	*P* values	AOR^b^ (95% CI) Model 2	*P* values	AOR (95% CI) Model 3	*P* values
Sex						
Male	Reference	—^c^	—	—	—	—
Female	1.759 (1.105‐2.802)	.02	—	—	—	—
Waist circumference (cm)						
Normal	Reference	—	Reference	—	Reference	—
High	2.33 (1.178‐4.618)	.02	0.95 (0.43‐2.07)	.90	0.94 (0.43‐2.06)	.89
Obesity						
No	Reference	—	—	—	—	—
Yes	4.21 (2.576‐6.885)	<.001^d^	—	—	—	—
ALT^e^						
Normal	Reference	—	Reference	—	Reference	—
High	2.44 (1.091‐5.462)	.03	3.23 (1.30‐8.06)	.01	3.37 (1.33‐8.48)	.01
AST^f^						
Normal	Reference	—	Reference	—	Reference	—
High	1.88 (0.637‐5.567)	.25	1.64 (0.48‐5.5)	.42	1.55 (0.45‐5.30)	.48
HOMA-IR^g^						
Normal	Reference	—	Reference	—	Reference	—
High	7.61 (3.545‐16.339)	<.001	7.41 (3.19‐17.2)	<.001	8.11 (3.42‐19.25)	<.001
Lipid-lowering agents						
No	Reference	—	Reference	—	—	—
Yes	0.57 (0.326‐1.023)	.06	0.53 (0.28‐1.00)	.05	—	—
Aspirin						
No	Reference	—	Reference	—	—	—
Yes	0.52 (0.331‐0.844)	.008	0.61 (0.36‐1.03)	.07	—	—

a UOR: unadjusted odds ratio.

b AOR: adjusted odds ratio.

c —: not applicable

d *P*<.05 denotes statistical significance.

e ALT: alanine aminotransferase.

f AST: aspartate aminotransferase.

g HOMA-IR: homeostatic model assessment of insulin resistance.

In addition to the associations mentioned, the analysis reveals several other noteworthy findings regarding the relationship between biochemical findings and MASLD. When MASLD was compared with ALT, AST, GGT, HbA_1c_, and albumin levels (regardless of stage), no significant associations were found (*P*>.05). However, a higher proportion of patients with MASLD had significantly elevated levels of total cholesterol (*P*=.01), triglycerides (*P*=.03), and LDL (*P*=.04). In addition, there was no significant association between MASLD and vitamin D (*P*=.92) or vitamin B_12_ (*P*=.92) levels. Furthermore, HOMA-IR exhibited a significant association with MASLD, with many patients with high HOMA-IR having MASLD compared with those with normal or low HOMA-IR (*P*<.001). The analysis also showed that glucose level was not significantly associated with MASLD (correlation coefficient *r*=0.03, *P*=.63). However, insulin level exhibited a significant positive correlation with MASLD (*r*=0.24; *P*=.001), indicating that as insulin levels increased, the likelihood of having MASLD also increased.

## Discussion

### Principal Results and Comparison With Prior Work

MASLD poses a global health concern, with rising prevalence linked to obesity and diabetes [12]. Soto et al [15] show that it is a widespread contributor to chronic liver disease globally. In Saudi Arabia, MASLD cases are projected to surge to more than 12 million by 2030 [16]. A study by Alswat et al [10] shows that by 2030, the estimated MASLD cases in Saudi Arabia and the United Arab Emirates would be 12,534,000 and 372,000, respectively. Lack of diagnosis denies patients crucial interventions. MASLD increases mortality risk, with significant economic burdens. According to Eskridge et al [17], MASLD severity correlates with mortality risk; after 20 years, excess risk ranges from 10.7% (simple steatosis) to 49.4% (cirrhosis). There is a need for better identification and management, particularly by primary care practitioners, emphasizing screening in high-risk populations, such as the obese and diabetics. Our study elucidates associations between MASLD and T2DM, offering insights for clinical intervention. Comparison with existing literature enhances understanding and identifies avenues for further research.

Our study reported that the prevalence of MASLD is 54.5%. Previous studies conducted in Saudi Arabia showed the prevalence of MASLD to be 47.8% (2015) in Jazan [18], 72.7% (2018) in Abha [14]. A recent analysis reported that the MASLD prevalence in Saudi Arabia from 2012 to 2019 was 28% to 33% [16], where older adults (older than 20 years) have an increased prevalence of MASLD from 41% to 44% [16]. The Middle East and North Africa regions have been identified as a primary source of MASLD, with prevalence rates exceeding 40% in most Middle East and North African countries [19,20]. Yemen, Turkey, and Kuwait had the lowest age-standardized disability-adjusted life years rates in 2021, whereas Egypt, Qatar, and Saudi Arabia had the highest rates. According to the 2019 Global Burden of Disease data, the high prevalence of obesity and diabetes in Egypt and Saudi Arabia is responsible for the high burden of chronic liver diseases, including MASLD [21]. This pattern is consistent with this information. According to Alenezi et al [22], Saudi Arabia had one of the highest rates of MASLD in the Middle East and North African region due to its obesity rate surpassing the global average. These geographic disparities are likely to be influenced by lifestyle patterns, socioeconomic factors, and access to health care.

Notably, the sociodemographic profiles of the study participants reflect a typical distribution seen in populations with T2DM, with a slight predominance of females (154/292, 52.7%) and a majority in the middle to older age groups (41‐59 years old; 192/292, 65.8%). In contrast, previous studies by Meo et al [23] and Kautzky-Willer et al [24] showed that the prevalence is higher in males at 11.2% as compared with females at 9.19%. Globally, men are 40% more likely to have MASLD than 26% of women [25]. This could be due to men and women storing fat differently, with women storing more fat subcutaneously and men having more visceral fat. This excess fat exacerbates liver damage. Estrogen, which protects against NAFLD, may also contribute to these differences, making men and postmenopausal women more susceptible to liver damage [5].

The high prevalence of comorbidities, such as dyslipidemia (218/292, 74.7%) and hypertension (209/292, 71.6%), underscores the multifactorial nature of T2DM and its associated complications. Similarly, Katundu et al [26] showed that dyslipidemia with low-density lipoprotein cholesterol derangement was highly prevalent, especially in individuals with both diabetes mellitus and hypertension. Likewise, some studies have reported that the prevalence of hypertension in individuals with MASLD was 40%‐60% [3]. In contrast, a previous study conducted in Saudi Arabia shows lower prevalence of hyperlipidemia (41.7%), T2DM (35.3%), and hypertension (28.4%) [5]. These findings suggest that the comorbidities are increasing in the Saudi population; however, longitudinal studies are needed to confirm the relationship.

Interestingly, while most participants were nonsmokers, a significant proportion exhibited abdominal obesity and overweight or obesity, highlighting the importance of lifestyle factors in metabolic health. Comparing these findings with previous studies, the prevalence of comorbidities aligns with established literature on the burden of cardiometabolic diseases in populations with T2DM [26]. However, the relatively high proportion of individuals with abdominal obesity warrants further investigation into the impact of visceral adiposity on liver health, particularly in the context of MASLD. Kuang et al [27] showed that there is increased MASLD risk in females with abdominal obesity phenotypes.

Notably, laboratory findings revealed a mixed metabolic profile among participants, with varying degrees of liver enzyme abnormalities, glycemic control, lipid levels, and insulin resistance. While elevated liver enzymes were associated with MASLD, other traditional markers of liver function, such as GGT, did not show significant associations. Huang et al [28] showed that participants with MASLD and elevated liver enzymes were younger, more often they were males, smokers, and had higher metabolic markers.

In our study, we found that insulin resistance, as measured by HOMA-IR, emerged as a significant predictor of MASLD, indicating the intricate interplay between insulin sensitivity and liver fat accumulation. According to new diagnostic criteria, the HOMA-IR is included as a diagnostic feature for T2DM in patients with MASLD [29]. A study by Fujii et al [30] shows that HOMA-IR was an independent predictor of advanced fibrosis. Thus, comparing these results with existing literature, the association between insulin resistance and MASLD aligns with the pathophysiological mechanisms of MASLD [31]. Insulin resistance promotes hepatic lipid accumulation by impairing insulin-mediated suppression of lipolysis and promoting de novo lipogenesis, contributing to the development and progression of MASLD.

Furthermore, several sociodemographic and clinical factors were investigated for their association with MASLD, with WC and obesity emerging as significant predictors of MASLD risk and cardiovascular disease [32]. These findings underscore the central role of adiposity in the pathogenesis of MASLD, highlighting the importance of weight management interventions in patients with T2DM at risk of liver disease. In addition, gender disparities in MASLD prevalence were observed, with females exhibiting a higher risk compared with males (*P*=.02), consistent with previous studies. Similarly, a study by Lonardo et al [33] showed that MASLD is more prevalent in men than women. Male gender and metabolic factors independently predict MASLD. Menopause also increases the risk of MASLD. Comparing these results with existing literature, the association between obesity and MASLD is well-established, with visceral adiposity driving insulin resistance, hepatic lipid accumulation, and inflammation [33]. However, the observed gender disparity in MASLD prevalence warrants further investigation into the underlying hormonal and metabolic factors contributing to sex-specific differences in liver disease susceptibility.

Thus, our study aligns with existing literature on MASLD risk factors in T2DM, showing that obesity, insulin resistance, and dyslipidemia are predictors of MASLD. While our findings were consistent with previous findings, some results, like the insignificance of certain biomarkers, indicate the necessity for further investigations.

### Limitations

Several limitations include the reliance on cross-sectional data, which precludes causal inference, and potential selection bias due to the study’s hospital-based design. In addition, the lack of longitudinal follow-up limits the assessment of disease progression, and the study’s sample size may restrict generalizability to broader populations. During the data extraction, we did not extract actual values of the laboratory tests, except for glucose and insulin. The laboratory tests were categorized during data extraction based on predefined thresholds. It is therefore considered that converting continuous variables into categories has limitations, impacting statistical power and bias risk. Maintaining continuous variables offers a nuanced understanding of nonlinear relationships.

### Conclusions

Our study contributes to our understanding of the epidemiology and pathophysiology of MASLD in populations with T2DM. By comparing these findings with existing medical literature, we enhanced our knowledge of the risk factors and clinical implications of MASLD, guiding future research and clinical practice in the prevention and management of liver disease in patients with T2DM. The findings of our study have important clinical implications for the management of patients with T2DM at risk of MASLD. Health care providers should prioritize screening for liver disease in individuals with abdominal obesity, insulin resistance, and dyslipidemia, as these factors are strongly associated with MASLD risk. Multidisciplinary approaches that integrate diabetes care with liver health monitoring and interventions are warranted to optimize patient outcomes and reduce the burden of liver disease in populations with T2DM. Future research should focus on elucidating the mechanistic pathways linking metabolic dysfunction, adiposity, and liver injury in patients with T2DM. Longitudinal studies are needed to assess the impact of lifestyle interventions, pharmacotherapy, and metabolic surgery on MASLD progression and clinical outcomes. In addition, exploring the role of genetic, epigenetic, and environmental factors in MASLD pathogenesis may yield novel insights into disease mechanisms and therapeutic targets.

## Supplementary material

10.2196/77772 Multimedia Appendix 1 Questionnaire used for data collection.

## Abbreviations

ALT: alanine aminotransferase
AST: aspartate aminotransferase
GGT: gamma-glutamyl transferase
HbA_1c_: glycated hemoglobin
HOMA-IR: Homeostatic Model Assessment of Insulin Resistance
KSMC: King Saud Medical City
LDL: low-density lipoprotein
MASLD: metabolic dysfunction associated steatotic liver disease
NAFLD: nonalcoholic fatty liver disease
NASH: nonalcoholic steatohepatitis
T2DM: type 2 diabetes mellitus
WC: waist circumference
