# Supplementary material for: Prevalence and Risk Factors of Metabolic Dysfunction–Associated Steatotic Liver Disease in Patients With Type 2 Diabetes Mellitus at a Tertiary Center in Saudi Arabia: Cross-Sectional Questionnaire Study
Source: JMIR Diabetes. 2025 Nov 3;10:e77772. doi: 10.2196/77772 (PMC12582413; doi:10.2196/77772)
Supplement: Multimedia Appendix 1 [file diabetes-v10-e77772-s001.docx]

**Data Collection Form**

**Section A: personal and demographic features**

1- Age: (years)

• 30-40

• 41-59

• >60

2- Gender:

• Male

• Female

3- Marital status:

• Married

• Non married

• Divorced

• Widow /Widower

4- Education level:

• Illiterate

• Elementary school

• High school

• College

• Post graduate

5- Vaccination In The last 12 month

• Yes Name of Vaccine (S)

1) ………………………………

2)…………………………………………

3)……………………………….

• No

**Section B: Risk factors**

6- Are you physically active (Do you Exercise regularly)?

• Yes

• No

7- Do you have one of these conditions: (you can mark more than one)

• Diabetes Mellitus

• Hypertension

• Hypertriglyceridemia

• Hypocholesteremia

• Hypothyroidism

• Bronchial asthma

• Cardiac diseases

• Stroke

• Epilepsy

• Depression

• Others…………………

8- Diabetes duration:

• < 5 years

• > 5 years

9- Treatment of diabetes

• Diet and exercise

• Oral hypoglycemic drugs

• Oral hypoglycemic drugs + Insulin

• Insulin only

• Others: ____________

10- ON Antihypertensive medications:

- No
- Yes If yes duration …………………

Drug Class:

1- Diuretics

2- ACEI

3- ARBs

4- CCB

5- Others (……………………………………………………………..)

11- On Lipid lowering medications:

- Yes
- No

12- On Aspirin:

- Yes
- No

13- Smoking history:

- Non-smoker
- Ex-smoker
- Current smoker

**Section C: Personal and laboratory data**

**Waist circumference measurement**: ___________cm

**Body mass index** _________ kg/m2

**Laboratory test**

ALT _______________

AST _______________

GGT ___________________

Normal High Low ________________

HOMA-IR Homeostatic Model Assessment of Insulin Insulin ____________________

mU/Lit × Glucose =HOMA-IR _____________________

HbA1C _______________

Albumin ______________

Glucose level _______________

Total cholesterol _______________

Triglyceride ______________

Total cholesterol _______________

Vitamin D _______________

Vitamin B12 _______________

**Liver ultrasonography was done within 12 months:**

- Yes
- No

If not done, ordered by the research investigator

- Yes
- No

**Result for any ordered ultrasound as per research protocol:**

Normal _____________

Abnormal: _____________

NAFLD ______________

Gallbladder stone __________________

others: ………………………………………………….
